# Supplementary figures and images for: Understanding patterns of loneliness in older long-term care users using natural language processing with free text case notes
Source: PLoS One. 2025 Apr 2;20(4):e0319745. doi: 10.1371/journal.pone.0319745 (PMC11964460; doi:10.1371/journal.pone.0319745)

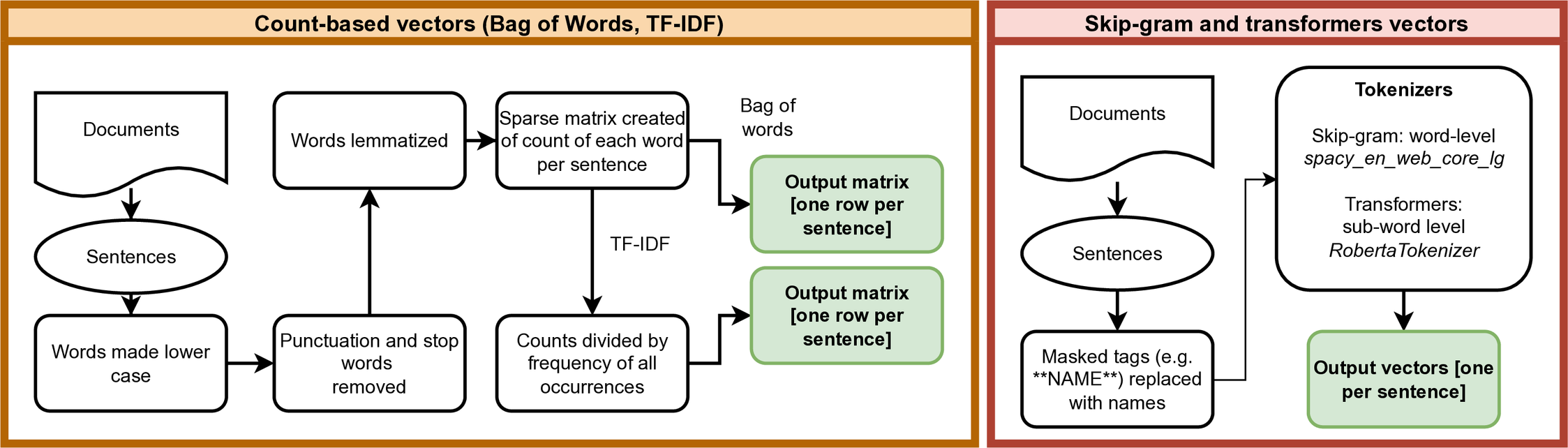

Supplement: S1 Fig — (TIF) [file pone.0319745.s001.tif]
