# Supplementary material for: Understanding patterns of loneliness in older long-term care users using natural language processing with free text case notes
Source: PLoS One. 2025 Apr 2;20(4):e0319745. doi: 10.1371/journal.pone.0319745 (PMC11964460; doi:10.1371/journal.pone.0319745)
Supplement: S1 Text — (PDF) [file pone.0319745.s003.pdf]

Using natural language processing to understand loneliness in older social care users from free text case notes: Supporting Information

Sam Rickman Jose-Luis Fernandez Juliette Malley

Appendices

1. S1 Data flow appendix.
2. S2 Methods appendix.
3. S3 GitHub repository.
4. S4 Additional results.

S1 Data Flow Appendix

In Figure 1, we set out the secure flow of identifiable data, the pseudonymisation process, data analysis and model output.

Figure 1: Data flow diagram

S2 Methods Appendix

We include below more details about the steps set out in the Model development section.

Data pre-processing

We received the free text with tokens in place of pseudonymised text, e.g. `**NAME**` or `**DATE**`. For count-based vectors, we pre-processed text by converting to lower case, removing stop words, such as "and" and "the" (Demner-Fushman, Chapman, and McDonald 2009). We also lemmatised words, restoring them to base form. For example, walks, walked and walking were all replaced with the normalised form, walk (Plisson et al. 2004) using the Python `NLTK` package's `WordNetLemmatizer` (Bird, Klein, and Loper 2009). We also replaced non-ASCII punctuation characters such as bullet points, which can cause text encoding issues.

Conversely, the word vector language representation models we used were trained on text without lemmatization, and including stop words, which optimises model performance (Alzahrani and Jololian 2021; Wang et al. 2020). However, we replaced masked text such as `**NAME**` or `**DATE**` with synthetic names and dates (using the same randomly chosen value for each tag) as set out in Table 1. We show in the example case note in our Methods and Materials section how, even in case notes which are indicative of loneliness or social isolation, much of the text in the note may be unrelated to the topic. To ensure that the classifier could learn which text is relevant, we split each case note into sentences for classification by human annotators, using the `SpaCy` Python package's sentence tokeniser (Honnibal and Montani 2021b). The data pre-processing is set out in Figure 2.

Table 1: Pseudonymisation masks

Mask	Replacement
**ETHNIC**	British
**EMAIL**	a@a.com
**NAME**	Byron
**POSTCODE**	SW1A 0AA
**DATETIME**	1970-01-01
**CURRENCY**	£
**TELEPHONE**	07777777777
**LOCATION**	London
**TIME**	3pm

Figure 2: Pre-processing

Labelling data in the training and test sets

The training and test sets each have over 300,000 sentences. It was neither possible nor desirable to annotate all case notes. Furthermore, as case notes can be about any topic, random selection would yield only a small proportion related to the area of interest. To maximise the number of relevant notes for the model to be trained on, we used active learning. This is a machine

learning “closed loop” method, where a seed set of dictionary terms is used to label a dataset, which is in turn used to train a classification model, which predicts more matches, which are in turn labelled, from which more terms are generated, until sufficient notes are annotated or saturation of the search terms is achieved (Yu et al. 2018). We began with a dictionary of terms such as “alone”, “isolated” and “friends”, selected to indicate sentences which *might* be related to social isolation and loneliness. Notes from the training set which contained these terms were then manually classified by human annotators. After the first set of notes were classified, we added terms from the classified notes to the list, until saturation was reached. We classified 10,083 sentences in the training set, and 3,573 sentences in the test set for model evaluation. We defined a set of rules for annotators to define which sentences to classify, using binary classification. Each note was annotated as being indicative of social isolation and loneliness (positive class), or not indicative (negative class). We include the initial dictionary, and classification rules, in the Classification rules section and the Dictionary terms section. It is important to note that the purpose of the dictionary was to prioritise sentences to be classified by human annotators: the final model can predict in either class sentences which do not contain these terms.

Both the training and test sets were manually classified by human annotators, using a binary classification scheme to identify sentences indicative of loneliness or social isolation. We measured inter-rater reliability using Cohen’s κ (McHugh 2012) and Krippendorf’s α (Krippendorff 2004). We randomly selected 300 sentences (150 in each class) to be classified by both human annotators. They agreed in 278 cases and disagreed in 22, giving κ of 0.89 (95% CI 0.84 - 0.94), and α of 0.89 (95% CI 0.89 - 0.93), calculated using the R `psych` and `krippendorffsalpha` packages (Revelle 2022; Hughes 2022). The maximum level of agreement in both cases is 1, and 0.89 represents excellent levels of agreement beyond chance (Fleiss, Levin, and Paik 1981; De Swert 2012).

Creating sentence vectors

Count-based and pre-trained vectors

Count-based approaches: document-term matrices and tf-idf. We split each sentence into lemmatised, word-level tokens as set out in Data pre-processing. We then created a document-term matrix. This is a large, sparse matrix where the columns are the set of all words in the corpus, and each row is represented by a sentence. The values in each row indicate the frequency of each word in the sentence (Nguyen 2013). We also applied tf-idf to transform the count matrix to a weighted representation, reducing the weighting of higher frequency words across all documents. We used the approach set out in (Pedregosa et al. 2011), where the tf-idf for a word w in a corpus c is,

$$\text{tf-idf}(w, c) = \text{tf}(w, c) \cdot \text{idf}(w)$$

$\text{tf}(w, c)$ is the count of the word in the corpus and $\text{idf}(w)$ is,

$$\text{idf}(w) = \log \frac{n}{\text{df}(w)} + 1$$

where n is the total number of documents in the corpus, and df(w) is the number of documents in the corpus containing the word w.

Pre-trained word embeddings We used the Spacy large English model (Honnibal and Montani 2021a). This model is trained on a large body of news, Wikipedia and subtitles data, and contains a parser based on OntoNotes 5 (Weischedel et al. 2013). The model represents language through dense embeddings (Mikolov et al. 2013), where words which have similar semantic meanings are clustered together in vector space. The model contains 684,830

unique, 300-dimensional pre-trained English vectors. We split each sentence into n word-level tokens, mapping each token to its corresponding vector. As sentence lengths differ, this creates variable-length representations of each sentence, which is a problem for many classification algorithms. Sentence embeddings constructed from an average of word embeddings have proven to be a strong baseline across a variety of tasks (Kenter, Borisov, and De Rijke 2016). We therefore stacked n token vectors for each sentence into a $300 \times n$ matrix, taking the mean of each dimension to create a single 300-dimensional vector to represent the sentence.

Transformer-based approaches

A transformer is a neural network architecture for computing representations of a sequence, which in our case is a sentence. Unlike the architecture used to generate the Spacy model (Honnibal and Montani 2021a), transformer-based approaches can learn context-dependent representations of words. Transformers use an attention mechanism to assign weights to each token in the sequence, aiming to allow the model to learn relevant long-range dependencies between tokens without the computational cost of calculating weights for all words in between (Vaswani et al. 2017). This avoids vanishing gradient problems that can arise in other architectures such as Recurrent Neural Networks (RNNs) as the size of the context window increases (Dehghani et al. 2018). Bidirectional Encoder Representations from Transformers (BERT) is a transformer-based model designed to be a general language representation that can be fine-tuned with just one additional layer to provide state-of-the-art models for a wide range of tasks, without significant task- or language-specific changes to the model's architecture (Devlin et al. 2018). For our task, we used RoBERTa, an incremental improvement on BERT with the same architecture but different training data and hyperparameters, and generally slightly improved accuracy (Liu et al. 2019). We used the RoBERTa *base* model, which has 12 hidden layers, 768 dimensions and 12 heads. This was relatively computationally expensive to fine-tune, so for comparison we also used DistilRoBERTa, which has identical parameters

except it has 6 hidden layers, and is around twice as fast to train. In both cases, we used the HuggingFace implementation of each model's tokeniser to split each sentence into sub-word tokens (Huggingface 2022a, 2022b). Each token was then converted by the model into a dense vector representation, based on pre-trained word embeddings, and further fine-tuned by the model to capture semantic information about the contextual meaning. These token-level vectors were then passed into the model's encoder, a neural network which produces a fixed-length vector representation of the input text, summarising the relevant information in the text for the model's classifier.

Classification algorithms for count-based and pre-trained vectors

The count-based and SpaCy approaches are methods to represent sentences in vector space. Once the representation of each sentence is in place, there are many methods that could reasonably be attempted to optimally distinguish the classes. In contrast, the transformer-based models we used (RoBERTa and DistilRoBERTa) inherently generate rich, contextual embeddings of text. For classification tasks, these embeddings are passed to a feed-forward neural network classifier appended as the final layer of the model. This classification layer is part of the standard implementation of transformer-based sequence classification models, such as those provided by the Hugging Face library, and was used in our study without modification. After pre-processing, vectorizing and labelling each sentence, the problem is a binary classification task. For both the count and pre-trained embedding based approaches, we evaluated five classification algorithms. We used k fold cross-validation to avoid overfitting on the training set, choosing 5 folds for k as a value which tends to elicit reasonably high accuracy (Nti, Nyarko-Boateng, and Aning 2021) while reducing training time compared with higher values.

The document-term matrix and tf-idf approaches both create sparse $m \times n$ matrices, where m

is the number of sentences and n is the number of unique terms (in our case $n = 5828$). To ensure that sparse matrices did not inhibit model performance, we reduced these to $m \times 1000$ matrices, using the implementation of truncated singular value decomposition in (Pedregosa et al. 2011), based on the method in (Halko, Martinsson, and Tropp 2009). However, this did not lead to improved classification, and the results in the Results section use the sparse matrices as inputs.

For the count-based and SpaCy vectors, we used the following classification algorithms:

1. Class-weighted logistic regression. We adjusted the C penalty parameter (weight of each class) in inverse proportion to the class frequency, so mistakes from the minority class have a higher cost, as in (Chaichulee et al. 2022). This was implemented using scikit-learn's built-in `class_weight="balanced"` setting.
2. Bootstrap aggregation ("bagging"). Bagging is one of the most effective classification algorithms for high-dimensional datasets (Bühlmann and Yu 2002). We used a decision-tree based bagging method, where multiple decision trees are created and trained on a bootstrapped sample of points drawn from a subset of the data. Once the decision trees have been trained, an overall prediction is created by aggregating the predictions and taking an average. The use of bootstrapped subsets of points and average predictions aim to reduce overfitting. We took an average of 10 decision tree classifiers, and did not limit the depth of the decision trees.
3. Random forest. This is a decision-tree approach where M decision trees are created to classify the data (Biau and Scornet 2016). As with bagging, each decision tree in the forest is created from a bootstrapped subset of the training data. However, in addition, each tree only uses a random subset of features (in our case words) from those points. This is done to prevent any individual decision tree from becoming too dependent on a single feature. Random forests can handle datasets with many features and are robust to

outliers. We set $M = 100$ trees.

4. Quadratic Discriminant Analysis (QDA). This is similar to Linear Discriminant Analysis (LDA), but allows each class to have its own covariance matrix (Tharwat 2016). The prior probability of each class is set to the proportion of training samples in each class. The likelihood of a novel data point being in each class is calculated from its position in vector space given the class-specific mean and covariance, and then multiplied by the prior class probabilities. The final prediction is the class which maximises this posterior probability.
5. Feed forward neural network. We tested with a range of hyperparameters to find the optimal architecture. The best performing network had three dense layers with 100 neurons, one drop-out layer and a final layer with one neuron (either 0 or 1 for the respective classes). In order to mitigate training problems caused by imbalanced classes, we used the same approach as with logistic regression, setting class weights in inverse proportion to their frequency. In addition, we tried oversampling the minority class to increase the number of positive samples available in the model, and undersampling the negative sentences. However, neither of these approaches was effective at improving metrics and the values in the Results section do not use these approaches.

We used the implementation in the Python Scikit-Learn package for each of these approaches (Pedregosa et al. 2011). We also replicated the feed forward neural network using TensorFlow in order to implement class weighting and over- and under-sampling (Abadi et al. 2015).

Classification metrics

We use the evaluation metrics for binary classification models set out in (Raschka and Mirjalili 2019), specifically accuracy, precision (positive predictive value), recall (true positive rate) and F1 score.

$$\text{accuracy} = \frac{TP + TN}{n}$$

$$\text{precision} = \frac{TP}{TP + FP}$$

$$\text{recall} = \frac{TP}{TP + FN}$$

$$F_1 = 2\frac{\text{precision} \cdot \text{recall}}{\text{precision} + \text{recall}}$$

Where n is the number of sentences classified, TP the number of true positives, FP false positives and FN false negatives. True negatives are included in accuracy, but not precision, recall or F_1. As the majority class is the negative class, it would be possible to achieve accuracy > 0.9 with a classifier which predicted that every sentence was in the negative class. We therefore use F_1 as the primary measure, which is the standard in binary classification tasks (see e.g. Chaichulee et al. 2022; Zhu et al. 2019)

Classification rules

This section contains the classification rules for manual annotators of the free text notes. We set out in Table 2 the rules for annotators to identify cases where loneliness or social isolation is indicated, and in Table 3 cases where it is not.

Lonely or socially isolated

Table 2: Loneliness or social isolation: positive cases

Situation	Example
Statement that the person is lonely and socially isolated	Mrs Byron appears lonely and has little social contact.
Statement the person feels lonely even if not socially isolated	She further advised that she gets out regularly and doesn't know why she feels lonely.
Statement that the person is socially isolated provided it does not state that they are not lonely	He does not go out or have any family or friends.
Statement that social isolation is a risk	High risk of social isolation.
Statement that person lives alone if they do not like it	Due to Mrs Byron being alone in the day she would like to request an increase.
Person expresses interest in attending a day centre with no reasons, or because of social isolation / loneliness. If reasons given it must be for social reasons (not for carer respite or managing safety).	She would like to attend the day centre. She would like to attend the day centre to increase social contact.
Referrals to befriending service	Byron stated that she did not need a carer but would like a befriender.
Receipt of befriending service	Byron's has a befriender who visits twice a week
Request for social support outside day centre	He said he does not want to go to a day centre but would like someone to talk to a couple of times a week.
Number of social contacts defined with subjective language	He barely sees family or friends.

Not lonely or socially isolated

Table 3: Loneliness or social isolation: negative cases

Situation	Example
Need for social support for purposes of managing safety	He attends a day centre on Mondays and Thursdays while his daughter is at work, as he cannot safely be left alone.
Need for social support for purposes of carer respite	He goes to a day centre two days a week to give his wife a break.
Need for support because of risks associated with safety	She needs support of one person outdoors because she is unable to safely assess risk crossing the road.
Need for support to manage practical tasks	He needs support with managing nutrition and meals.
Generic request for support	She has requested social services support as she is anxious re ability to cope.
Need for support because of depression/anxiety	She has requested social services support, as she informed me that her mother appeared to be depressed , staying in her bed all the time.
Person is isolated in the sense of infection-control.	He has been isolated in his room until he does not have a fever.
Statement that person lives alone with no additional information	He lives alone
Person has n social contacts per week without subjective language	He seems family twice a week.
The fact that a person attends a day centre	Mr Byron has a very social active life, he attends Day Centres and Clubs between Monday to Friday.
Offer of day centre refused	I offered to refer Mrs X to a day centre but she declined.
Befriending for the purpose of carer support	I will also refer to AGE UK Clinical navigators for any assistance they may be able to provide such as a sitting service/befriending to allow Mrs X time to attend her own hospital appointments.

Note on befriending and day centre services

Although befriending and day centres are both services for loneliness, they are treated differently in the following way. The assumption is that as befriending is a voluntary service with a high threshold and low intensity (often an hour visit per week), individuals receiving it may not receive sufficient quantity to eliminate loneliness. Conversely, day centre services have a much higher intensity, typically at least several hours per day. While we assume that at the point of day centre referral there is a need for social inclusion (unless the referral is for other reasons such as carer respite), we do not assume that all individuals in receipt of day services continue to be lonely or socially isolated. This is set out in Table 4.

Table 4: Loneliness or social isolation: befriending vs day centre

	Point of referral	Service currently being received
Befriending	Lonely	Lonely
Day centre	Lonely	Not lonely

Wide dictionary terms

This section contains the dictionary terms used to prioritise case notes to be classified by humans. It is important to note that the purpose of the dictionary was to prioritise sentences to be classified by human annotators. Sentences that contain these terms are not automatically classified by the model as indicative of loneliness or social isolation. Similarly, sentences which the model classifies as indicative of loneliness or isolation do not need to contain any of these terms.

```
alone
companionship
divorce
divorced
engagement
family support
friends
isolated
isolation
left out
lives alone
loneliness
lonely
lonesome
no friend
no friends
on her own
on his own
on their own
reclusive
secluded
separated
single
social connection
social contact
social network
social support
social withdraw
socially isolating
```

```
socially withdraw
solitary
solitude
support network
widow
widowed
withdrawn
befriend
interaction
activities
company
bored
```

S3 Open source version of model

Our classification model can be downloaded and run at https://github.com/samrickman/lonelinessmodel, which can be run on large volumes of free text to generate classifications. The model is reproducible as it is encapsulated in a Docker container. This has been archived on Zenodo: https://doi.org/10.5281/zenodo.13934375.

S4 Additional results

Table of results

We set out in Table 5 the data represented in the table in Figure 4. Proportion of lonely/isolated by demographic characteristics: administrative and survey data.

Table 5: Comparison of free text extraction with ELSA

		ELSA		Administrative data	
Group	Category	CES	UCLA	Assessment	Case notes
Age	<82	0.32 (0.27 - 0.36)	0.4 (0.33 - 0.48)	0.4 (0.36 - 0.44)	0.42 (0.38 - 0.46)
Age	82+	0.44 (0.34 - 0.54)	0.5 (0.42 - 0.58)	0.46 (0.42 - 0.5)	0.46 (0.43 - 0.5)
Gender	F	0.38 (0.32 - 0.45)	0.46 (0.4 - 0.52)	0.45 (0.41 - 0.48)	0.44 (0.41 - 0.48)
Gender	M	0.37 (0.28 - 0.46)	0.43 (0.31 - 0.55)	0.41 (0.37 - 0.45)	0.45 (0.4 - 0.49)
Ethnicity	Non-white	0.48 (0.3 - 0.66)	0.39 (0.13 - 0.66)	0.42 (0.38 - 0.47)	0.43 (0.39 - 0.48)
Ethnicity	White	0.38 (0.32 - 0.44)	0.45 (0.39 - 0.52)	0.44 (0.4 - 0.47)	0.45 (0.42 - 0.48)
Lives alone	No	0.23 (0.19 - 0.27)	0.32 (0.26 - 0.39)	0.4 (0.36 - 0.44)	0.41 (0.37 - 0.45)
Lives alone	Yes	0.5 (0.41 - 0.59)	0.56 (0.46 - 0.65)	0.46 (0.42 - 0.5)	0.47 (0.44 - 0.51)
Dressing	Independent	0.36 (0.3 - 0.43)	0.42 (0.35 - 0.5)	0.49 (0.43 - 0.54)	0.5 (0.44 - 0.56)
Dressing	Requires support	0.4 (0.32 - 0.49)	0.5 (0.43 - 0.56)	0.42 (0.39 - 0.45)	0.43 (0.4 - 0.46)
Toileting	Independent	0.36 (0.3 - 0.41)	0.43 (0.37 - 0.49)	0.43 (0.39 - 0.47)	0.49 (0.45 - 0.53)
Toileting	Requires support	0.52 (0.44 - 0.61)	0.59 (0.51 - 0.66)	0.44 (0.4 - 0.47)	0.4 (0.37 - 0.44)
Mobility	Independent	0.34 (0.21 - 0.46)	0.37 (0.2 - 0.55)	0.48 (0.43 - 0.52)	0.51 (0.46 - 0.55)
Mobility	Requires support	0.39 (0.33 - 0.45)	0.47 (0.4 - 0.53)	0.41 (0.38 - 0.44)	0.41 (0.37 - 0.44)
Meals	Independent	0.34 (0.29 - 0.39)	0.42 (0.36 - 0.48)	0.46 (0.38 - 0.55)	0.5 (0.42 - 0.59)
Meals	Requires support	0.54 (0.41 - 0.67)	0.6 (0.5 - 0.69)	0.43 (0.4 - 0.46)	0.44 (0.41 - 0.47)
Shopping	Independent	0.3 (0.25 - 0.35)	0.37 (0.31 - 0.43)	0.3 (0.18 - 0.45)	0.45 (0.31 - 0.61)
Shopping	Requires support	0.49 (0.4 - 0.59)	0.57 (0.49 - 0.65)	0.44 (0.41 - 0.46)	0.44 (0.42 - 0.47)
Memory	Independent	0.38 (0.32 - 0.44)	0.46 (0.39 - 0.52)	0.33 (0.29 - 0.37)	0.35 (0.31 - 0.39)
Memory	Requires support	0.44 (0.073 - 0.81)	0.3 (-0.015 - 0.61)	0.5 (0.46 - 0.53)	0.5 (0.47 - 0.54)

References (appendices)

Abadi, Martín, Ashish Agarwal, Paul Barham, Eugene Brevdo, Zhifeng Chen, Craig Citro, Greg S. Corrado, et al. 2015. "TensorFlow: Large-Scale Machine Learning on Heterogeneous Systems." https://www.tensorflow.org/.

Alzahrani, Esam, and Leon Jololian. 2021. "How Different Text-preprocessing Techniques Using The BERT Model Affect The Gender Profiling of Authors." *arXiv Preprint arXiv:2109.13890*.

Biau, Gérard, and Erwan Scornet. 2016. "A random forest guided tour." *Test* 25: 197–227.

Bird, Steven, Ewan Klein, and Edward Loper. 2009. *Natural language processing with Python: analyzing text with the natural language toolkit*. " O'Reilly Media, Inc.".

Bühlmann, Peter, and Bin Yu. 2002. "Analyzing bagging." *The Annals of Statistics* 30 (4): 927–61.

Chaichulee, Sitthichok, Chissanupong Promchai, Tanyamai Kaewkomon, Chanon Kongkamol, Thammasin Ingviya, and Pasuree Sangsupawanich. 2022. "Multi-label classification of symptom terms from free-text bilingual adverse drug reaction reports using natural language processing." *PloS One* 17 (8): e0270595.

De Swert, Knut. 2012. "Calculating inter-coder reliability in media content analysis using Krippendorff's Alpha." *Center for Politics and Communication* 15.

Dehghani, Mostafa, Stephan Gouws, Oriol Vinyals, Jakob Uszkoreit, and Łukasz Kaiser. 2018. "Universal transformers." *arXiv Preprint arXiv:1807.03819*.

Demner-Fushman, Dina, Wendy W Chapman, and Clement J McDonald. 2009. "What can natural language processing do for clinical decision support?" *Journal of Biomedical Informatics* 42 (5): 760–72.

Devlin, Jacob, Ming-Wei Chang, Kenton Lee, and Kristina Toutanova. 2018. "Bert: Pre-training of deep bidirectional transformers for language understanding." *arXiv Preprint arXiv:1810.04805*.

Fleiss, Joseph L, Bruce Levin, and Myunghee Cho Paik. 1981. "The measurement of interrater agreement." *Statistical Methods for Rates and Proportions* 2 (212-236): 22–23.

Halko, Nathan, Per-Gunnar Martinsson, and Joel A Tropp. 2009. "Finding structure with randomness: Probabilistic algorithms for constructing approximate matrix decompositions arXiv [math. NA]."

Honnibal, M., and I. Montani. 2021a. "English pipeline optimized for CPU. Components: tok2vec, tagger, parser, senter, ner, attribute ruler, lemmatizer." https://github.com/explosion/spacy-models/releases/tag/en_core_web_lg-3.0.0.

———. 2021b. "spaCy 3: Natural language understanding with Bloom embeddings, convolutional neural networks and incremental parsing." https://spacy.io/.

Huggingface. 2022a. "distilroberta-base." https://huggingface.co/distilroberta-base.

———. 2022b. "roberta-base." https://huggingface.co/roberta-base.

Hughes, John. 2022. *krippendorffsalpha: Measuring Agreement Using Krippendorff's Alpha Coefficient*. https://CRAN.R-project.org/package=krippendorffsalpha.

Kenter, Tom, Alexey Borisov, and Maarten De Rijke. 2016. "Siamese cbow: Optimizing word embeddings for sentence representations." *arXiv Preprint arXiv:1606.04640*.

Krippendorff, Klaus. 2004. "Measuring the reliability of qualitative text analysis data." *Quality and Quantity* 38: 787–800.

Liu, Yinhan, Myle Ott, Naman Goyal, Jingfei Du, Mandar Joshi, Danqi Chen, Omer Levy, Mike Lewis, Luke Zettlemoyer, and Veselin Stoyanov. 2019. "Roberta: A robustly optimized bert pretraining approach." *arXiv Preprint arXiv:1907.11692*.

McHugh, Mary L. 2012. "Interrater reliability: the kappa statistic." *Biochemia Medica* 22 (3): 276–82.

Mikolov, Tomas, Kai Chen, Greg Corrado, and Jeffrey Dean. 2013. "Efficient estimation of word representations in vector space." In *International Conference on Learning Representations (ICLR)*.

Nguyen, Eric. 2013. "Text mining and network analysis of digital libraries in R." *Data Mining Applications with R*, 95–115.

Nti, Isaac Kofi, Owusu Nyarko-Boateng, and Justice Aning. 2021. "Performance of Machine Learning Algorithms with Different K Values in K-fold Cross-Validation." *International Journal of Information Technology and Computer Science* 13 (6): 61–71.

Pedregosa, F., G. Varoquaux, A. Gramfort, V. Michel, B. Thirion, O. Grisel, M. Blondel, et al. 2011. "Scikit-learn: Machine Learning in Python." *Journal of Machine Learning Research* 12: 2825——2830.

Plisson, Joël, Nada Lavrac, Dunja Mladenic, et al. 2004. "A rule based approach to word lemmatization." In *Proceedings of IS*, 3:83–86. Citeseer.

Raschka, Sebastian, and Vahid Mirjalili. 2019. *Python machine learning: Machine learning and deep learning with Python, scikit-learn, and TensorFlow 2*. Packt Publishing Ltd.

Revelle, William. 2022. *psych: Procedures for Psychological, Psychometric, and Personality Research*. Evanston, Illinois: Northwestern University. https://CRAN.R-project.org/package=psych.

Tharwat, Alaa. 2016. "Linear vs. quadratic discriminant analysis classifier: a tutorial." *International Journal of Applied Pattern Recognition* 3 (2): 145–80.

Vaswani, Ashish, Noam Shazeer, Niki Parmar, Jakob Uszkoreit, Llion Jones, Aidan N Gomez, Łukasz Kaiser, and Illia Polosukhin. 2017. "Attention is all you need." *Advances in Neural Information Processing Systems* 30.

Wang, Wenhui, Furu Wei, Li Dong, Hangbo Bao, Nan Yang, and Ming Zhou. 2020. "Minilm: Deep self-attention distillation for task-agnostic compression of pre-trained transformers." *Advances in Neural Information Processing Systems* 33: 5776–88.

Weischedel, Ralph, Martha Palmer, Mitchell Marcus, Eduard Hovy, Sameer Pradhan, Lance Ramshaw, Nianwen Xue, et al. 2013. "Ontonotes release 5.0 ldc2013t19." *Linguistic Data Consortium, Philadelphia, PA* 23.

Yu, Hualong, Xibei Yang, Shang Zheng, and Changyin Sun. 2018. "Active learning from imbalanced data: A solution of misc weighted extreme learning machine." *IEEE Transactions on Neural Networks and Learning Systems* 30 (4): 1088–1103.

Zhu, Vivienne J, Leslie A Lenert, Brian E Bunnell, Jihad S Obeid, Melanie Jefferson, and Chanita Hughes Halbert. 2019. "Automatically identifying social isolation from clinical narratives for patients with prostate Cancer." *BMC Medical Informatics and Decision Making* 19 (1): 1–9.
